# Epidemiology of Guillain-Barré Syndrome in Iranian Children Aged 0-15 Years (2008-2013)

**DOI:** 10.22037/ijcn.v15i4.25087

**Published:** 2021

**Authors:** Seyed Hassan TONEKABONI, Sussan MAHMOUDI, Fatemeh ABDOLLAH GORJI, Habibe NEJAD BIGLARI, Mohammad Mahdi TAGHDIRI, Koroush ETEMADI, Mohammad GHOFRANI, Abdollah KARIMI, Mohammad REZAEI ZADEH

**Affiliations:** 1 Pediatrics Neurology Research Center, Shahid Beheshti University of Medical Sciences, Tehran, Iran.; 2 National polio Eradication Focal Point, Ministry of Healthand Medical Education, Tehran,Iran; 3 Clinical Research Development Center, MofidChildren’s Hospital, ShahidBeheshti University of MedicalSciences, Tehran, Iran.; 4 Pediatric Neurology Department, Kerman University of Medical Sciences,Kerman, Iran.; 5 5.Environmental and Occupational Hazards Control Research Center, Department of Epidemiology, Faculty of Health,Shahid Beheshti University of Medical Sciences, Tehran, Iran.; 6 Pediatrics Infectious Research Center, Shahid Beheshti University of Medical Sciences, Chairman of National Certification Committee for Polio Eradication, Tehran, Iran.; 7 Student Research Committee, School of Medicine, Kerman University of Medical Sciences, Kerman, Iran

**Keywords:** Guillain-Barré Syndrome, Incidence, Children, Epidemiology, Iran

## Abstract

**Objective:**

Guillain-Barré Syndrome (GBS) is an acute inflammatory polyneuropathy characterized by a rapid progressive symmetric weakness. The GBS is the most common cause of acute flaccid paralysis (AFP) in most parts of the world. This study was carried out to investigate the epidemiological features of GBS in Iranian children.

**Materials & Methods:**

The data were extracted using the AFP surveillance system that is a national screening program to detect all cases of AFP aged 0-15 years around the country. National Population Statistics data and AFP demographic data during 2008-2013 intervals were obtained from the relevant authorities in the Ministry of Health in Iran. The GBS cases were then extracted from the aforementioned database. The Chi-square test and Fisher’s exact test were used for statistical analysis.

**Results:**

A total of 1884 cases of GBS were identified in the study period, and the average annual incidence rate was 1.72 per 100,000 individuals. The highest incidence rate was within the range of 0-5 years. There was no statistically significant relationship between the incidence of GBS and the season in the whole country.

**Conclusion:**

High costs of GBS treatment, morbidity and occasional mortality, and number of new cases, which is estimated to be approximately 300 individuals per year, need the particular attention of the health system.

## Introduction

Guillain-Barré Syndrome (GBS) is an acute acquired peripheral neuropathy characterized by rapidly developing motor weakness (1). The GBS has an autoimmune etiology and in the two-third of cases is triggered by respiratory or gastrointestinal infections (2, 3). The pathology is an inflammatory disorder affecting the myelin sheath and the axon leading to diffuse demyelination and axonal degeneration (4).

The GBS is the most common cause of subacute and acute flaccid paralysis (AFP) in neonates and children. All age groups can be affected by GBS, and it seems that adults are more susceptible than children. The worldwide incidence of GBS for all ages has been estimated to be within the range of 0.4-2.4 per 100,000 individuals. The annual incidence in children seems to be lower estimated to be within the range of 0.4-1.3 per 100,000 individuals. 

The prognosis of GBS in children is generally excellent that is quite different from adults who have higher morbidity and mortality (4). There is no significant difference in GBS incidence among seasons, although a limited number of studies suggested a seasonal variation (1). With this background in mind, the current study aimed to determine the annual incidence of GBS in children, analyze morbidity and mortality rates, and compare these rates according to age, gender, and geographical and seasonal variations.

## Materials & Methods

This cross-sectional study was conducted on Iranian children under 15 years of age with GBS within 2008 and 2013. Patients’ information was obtained using the database extracted from the AFP surveillance system, which is designed by the World Health Organization (WHO) to screen children aged 0-15 years with AFP in polio-free countries with a high risk of imported polio. This program is implemented by the Preventable Diseases Department of the Ministry of Health in Iran and is annually evaluated and approved by the WHO. 

These AFP cases are classified in each province by regional AFP committees (composed of infection, epidemiologist, and neurologist experts). Then, GBS patients were separated from other AFP cases and analyzed. The GBS children information sheet included patients’ code, name, age, gender, date of birth, date of paralysis, early-onset symptoms of the disease, a remnant of paralysis after 60 days of occurrence, and home address (within 2008 and 2013). 

Based on the 2008 census and demographic estimates, the populations of each province and the whole country were obtained from the Statistics National Center for the years 2008-2013. Total Iranian populations were 70,495,882 and 75,149,669 individuals after the 2006 and 2011 censuses, respectively. The numbers of children under 15 years of age were 17,681,629 and 17,561,778 in 2006 and 2011, respectively. 

The data were collected from information sheets and entered in SPSS software (version 20) and analyzed. Mean and standard deviation and relative and absolute frequency were used to analyze quantitative and qualitative variables, respectively. Moreover, the Chi-square test and Fisher’s exact test were performed to investigate the association between demographic and epidemiological factors with GBS. A p-value of less than 0.05 was considered statistically significant. 

## Results

Within 2008 and 2013, 1884 cases of GBS were diagnosed, including 333 cases in 2008, 309 cases in 2009, 322 cases in 2010, 307 cases in 2011, 321 cases in 2012, and 292 cases in 2013 with the mean age values of 4.96±3.54, 5.60±3.96, 5.47±3.77, 5.66±3.97, 5.25±3.54, and 6.05±3.79 years, respectively. Altogether, 1061 (56.3%) and 823 (43.7%) children were male and female, respectively. Furthermore, there was no statistically significant difference between the genders of patients in each year (P=0.587).

**Fig 1:**
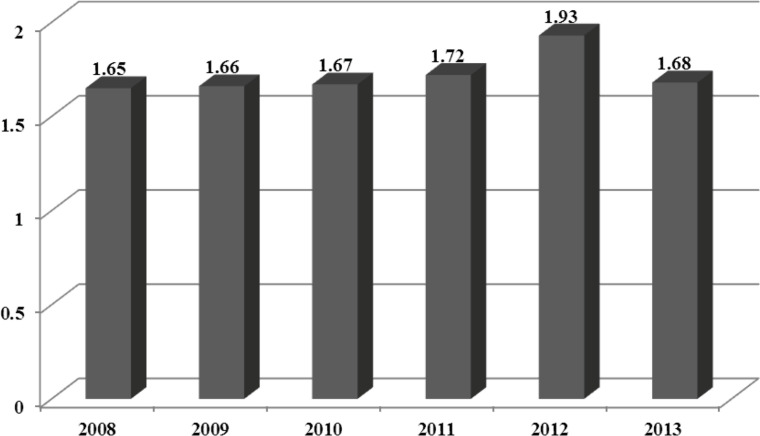
Guillain-Barre syndrome incidence in children under 15 years old during 2008-13

The mean incidence rate of GBS during the study period (2008-2013) was 1.72 per 100,000 individuals in children under 15 years of age. The highest (1.93%) and lowest (1.65%) incidence rates were reported in 2012 and 2008, respectively (Figure 1). Overall, during the study, the investigation of the age distribution of cases showed 4% of cases under 1 year, 50% of cases within 1-5 years, 30% of cases within 5-10 years, and 16% of cases within 10-15 years of age.

In this study, 579 (30.7%) cases had a fever during the last week before paralysis. Residual motor deficit after 60 days of paralysis onset was in the order of 31.8% in 2008, 34.4% in 2009, 35.1% in 2010, 34.0% in 2011, 27.1% in 2012, and 22.7% in 2013. The rest of the patients, excluding death cases, were recovered before 60 days. Residual paralysis or weakness (at least one muscle group at ≤ grade 6 level) ranged from steppage gait to non-ambulatory state (i.e., bed or chair bound). Grade 6 is the ability to move a muscle against gravity with minimal resistance to force.

Out of 1884 cases, 29 children (1.5%) with GBS died. The results also showed that there was a significant difference between the mortality rate and age group (P=0.024) with maximum mortality in children under 5 years of age; however, there was no significant difference between the mortality rate and the gender of children with GBS (P=0.451; Table 1)

**Table 1:** Age Distribution and Gender Influence on Guillain-Barré Syndrome Outcomes in Children Under 15 Years of Age

	Outcome n (%)				
	*Alive*	*Death*	*Full remission*	*Residual motor deficit*	
Gender					
*Female* *Male*	**809 (98.3)** **1046 (98.6)**	**14 (1.7)** **15 (1.4)**	**576 (67)** **729 (68.7)**		**248 (33)** **332 (31.3)**
Age (year)					
*<1 * *1-5 * *5-10* *10-15 *	**72 (96)** **922 (97.9)** **562 (99.3)** **299 (99.3)**	**3 (4)** **20 (2.1)** **4 (0.7)** **2 (0.7)**	**54 (72)** **649 (69)** **396 (70)** **206 (68.4)**		**21 (28)** **293 (31)** **170 (30)** **95 (31.6)**

**Fig 2:**
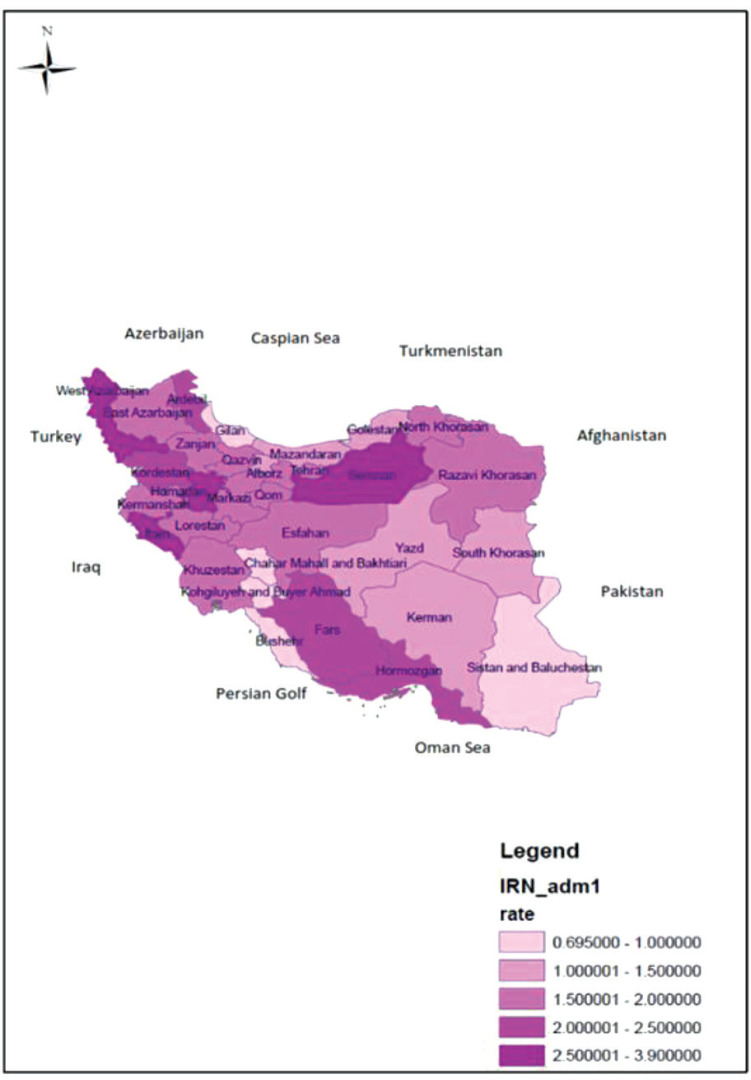
the distribution incidence rate of Guillain Barre Syndrome per 100,000 populations of children less than 15 years old in each province of Iran (2008-2013)

Figure 2 shows the distribution of incidence rates of GBS in different Iranian provinces. According to Figure 2, Semnan (3.7 in 100,000) and Ilam (2.8 in 100,000) have the highest incidence rates of GBS, and Bushehr (0.7 in 100,000) and Gilan (0.8 in 100,000) have the lowest incidence rates of GBS.

During 6 years of study (2008-2013), the incidence rates of GBS were 1.6 and 1.9 in girls and boys per 100,000 children under 15 years of age, respectively.

**Fig 3:**
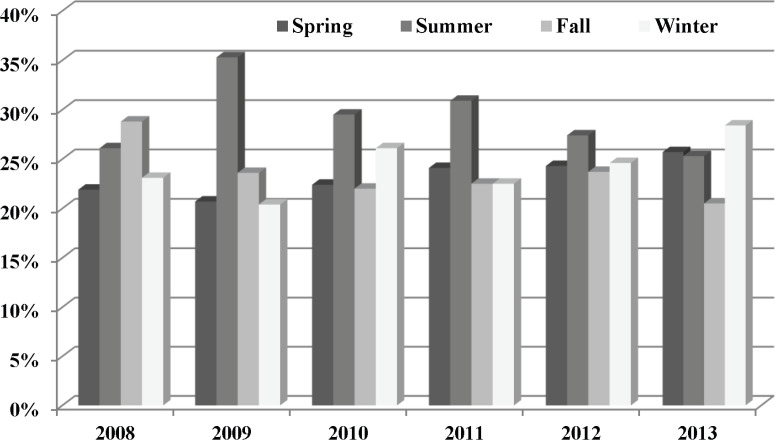
Seasonal variability of GBS incidence in children less than 15 years (2008-2013)

Figure 3 shows seasonal variability of GBS incidence in children illustrating the percentage of patients who were affected during each season based on the study years. The incidence rate of GBS according to the season during 2008-2013 showed no significant difference among the seasons in different years (P=0.157). 

## Discussion

This descriptive cross-sectional retrospective study was based on the data from the AFP surveillance system in Iranian children aged 0-15 years with GBS during 2008-2013 intervals. Historically, the overall incidence rate of GBS at all ages was within the range of 0.4/100,000 in Sardinia, Italy, (5) to 4/100,000 in Larimer County, Colorado (6). The incidence of GBS in children (age range: 0-15 years) seems to be lower than that in adults with a range of 0.34-1.34/100,000 per year (1). The highest rate was reported in adult patients above 75 years of age (4.5/100,000) (1).

With 1884 cases of GBS, the present study reported the largest number of patients aged 0-15 years from one country (1), preceded by olive et al. reporting 1678 affected children from Brazil (7). The prospective method of studying GBS incidence is considered to be more reliable, compared to the retrospective one (1). Nevertheless, in the current retrospective study, each case with AFP was reviewed by an expert committee and the diagnosis of GBS was confirmed, minimizing the risk of overestimation of GBS reports. Regarding two national censuses in 2006 and 2011, the total population of the present study is accurate. 

The case definition in the current study was based on AFP patients reported from all around the country. The GBS cases were identified after further clinical and electrophysiological studies; this means that the major manifestation was a motor deficit. In addition, theoretically, this study could have missed rare cases of acute sensory neuropathy with only sensory nerves or roots involvement and atypical cases, such as Miller Fisher syndrome that is not presented with flaccid paralysis. However, this fact can have a negligible effect since these atypical cases are rare (less than 5%), and they can only change the incidence of GBS by approximately 0.1 per 100.000 individuals (8, 9).

A recent study from Bangladesh showed an incidence rate of 1.5 to 2.5/100,000 individuals per year in children under 15 years of age (10), indicating a probable increase in childhood GBS incidence. With a mean incidence of 1.72 per 100,000 individuals, the present study supports the aforementioned hypothesis. This presumed rise in incidence could be explained by a better diagnosis of the disease by physicians, a better national registry network, and the emergence of many new environmental factors (i.e., chemical, physical, and microbiological) that can modulate the immune system. However, researchers should wait for performing extensive surveys from other countries to document the possible rise in GBS incidence.

In a 6-year cross-sectional study (2001-2006) in East Azerbaijan, Iran, Barzegar et al. reported an incidence of GBS, in a 0-to-15-year interval, ranging from 0.16 to 4 per 100.000 individuals (11). There was a peak incidence in 2003 (probably after a campylobacter outbreak in the northwest of Iran) with a similar peak in GBS mortality during this year). All studies showed a male predominance in GBS which is unusual in a presumed autoimmune disease. The male: female GBS ratios in all ages were reported as 1.25:1 in the UK (3), 1.7:1 in Western Balkans countries, and 1.1:1 in Spain (12). The male to female ratio was 1.27:1 in the present cohort that is almost similar to those of other countries in all age groups. 

Childhood GBS generally has a good prognosis in contrast to adult GBS with higher morbidity and mortality (4). The mortality rate in childhood GBS was 0% in Toronto, Canada (13); nonetheless, there was a mortality rate of 1.5% which could be explained by delay in patients’ referral to tertiary hospitals when sophisticated respiratory and intensive care unit care was available. During an 11-year study period (1989 -1999) in Honduras, the mortality rate in childhood GBS was 7.3%. Most of the mortality cases of the present study occurred in children under 5 years of age. The reason for this age distribution is the non-specificity of symptoms in early childhood, delay in the correct diagnosis, and vulnerability of young children to respiratory failure and autonomic dysfunction (14).

Residual motor deficit after 60 days of paralysis onset annually ranged from 22.7% to 35.1%. In this study, there was no longer follow-up for the patients. Vajsar J et al. reported a persistently decreased muscle power (with at least one muscle group having movement against gravity but with minimal resistance against a force) in 23% of children with GBS during an average 5-year follow-up in Toronto (13).

No significant seasonal variation in GBS incidence was reported in other studies (15-18), although a Brazilian study showed outbreaks in spring and summer (19) and another study in Netherland showed an increase in incidence in winter and June (20). Xiujuan Wu et al. reported a higher incidence of childhood GBS in summer (21). Despite different climates in Iran, there was no significant difference in the seasonal incidence of GBS in the patients of this study.

The age distribution of GBS is bimodal according to some studies, with peaks occurring in young adults and the elderly (2, 22, 23). In the current study, 50% of all GBS cases were within the age range of 1-5 years, for which there was no explanation and no similar results were obtained in other studies.
